# Cellular Factor XIII, a Transglutaminase in Human Corneal Keratocytes

**DOI:** 10.3390/ijms20235963

**Published:** 2019-11-27

**Authors:** Zsuzsanna Z. Orosz, Helga Bárdos, Amir H. Shemirani, Ildikó Beke Debreceni, Riitta Lassila, Antti S. Riikonen, Johanna A. Kremer Hovinga, Theo G. Seiler, Hendrika A. van Dorland, Verena Schroeder, Zoltán Boda, László Nemes, Beatrice Früh Eppstein, Bence Nagy, Andrea Facskó, János Kappelmayer, László Muszbek

**Affiliations:** 1 Division of Clinical Laboratory Science, Department of Laboratory Medicine, University of Debrecen, Faculty of General Medicine, 4032 Debrecen, Hungary; zsorosz@med.unideb.hu (Z.Z.O.); amir@med.unideb.hu (A.H.S.); 2 Department of Ophthalmology, University of Szeged, 6720 Szeged, Hungary; aafacsko@gmail.com; 3 Department of Preventive Medicine, University of Debrecen, 4028 Debrecen, Hungary; bardos.helga@sph.unideb.hu; 4 Department of Laboratory Medicine, University of Debrecen, Faculty of General Medicine, 4032 Debrecen, Hungary; ideb@med.unideb.hu (I.B.D.); kappelmayer@med.unideb.hu (J.K.); 5 Coagulation Disorders Unit, Department of Hematology, Comprehensive Cancer Center, University of Helsinki and Helsinki University Hospital, 00029 Helsinki, Finland; riitta.lassila@kolumbus.fi; 6 Department of Ophthalmology, University of Helsinki and Helsinki University Hospital, 00014 Helsinki, Finland; antti.riikonen@hus.fi; 7 Department of Hematology and Central Hematology Laboratory, Inselspital, Bern University Hospital, and Department for BioMedical Research, University of Bern, 3008 Bern, Switzerland; Johanna.Kremer@insel.ch (J.A.K.H.); anette.vandorland@insel.ch (H.A.v.D.); 8 Universitätsklinik für Augenheilkunde, Inselspital, Universität Bern, 3010 Bern, Switzerland; guentertheodormichael.seiler@insel.ch (T.G.S.); Beatrice.Frueh@insel.ch (B.F.E.); 9 Department for BioMedical Research (DBMR), Experimental Haemostasis Group, University of Bern, 3008 Bern, Switzerland; verena.schroeder@dbmr.unibe.ch; 10 Department of Internal Medicine, Thrombosis and Haemostasis Center, University of Debrecen, 4032 Debrecen, Hungary; zboda@med.unideb.hu; 11 National Haemophilia Center and Haemostasis Department, State Health Center, 1134 Budapest, Hungary; lnemes@t-online.hu; 12 Department of Pathology, University of Szeged, 6270 Szeged, Hungary; nagybencedr@gmail.com

**Keywords:** cornea, factor XIII, isopeptide bonds, keratocytes, transglutaminase

## Abstract

Cellular factor XIII (cFXIII, FXIII-A_2_), a transglutaminase, has been demonstrated in a few cell types. Its main function is to cross-link proteins by isopeptide bonds. Here, we investigated the presence of cFXIII in cells of human cornea. Tissue sections of the cornea were immunostained for FXIII-A in combination with staining for CD34 antigen or isopeptide cross-links. Isolated corneal keratocytes were also evaluated by immunofluorescent microscopy and flow cytometry. FXIII-A in the corneal stroma was quantified by Western blotting. FXIII-A mRNA was detected by RT-qPCR. The cornea of FXIII-A-deficient patients was evaluated by cornea topography. FXIII-A was detected in 68 ± 13% of CD34+ keratocytes. Their distribution in the corneal stroma was unequal; they were most abundant in the subepithelial tertile. cFXIII was of cytoplasmic localization. In the stroma, 3.64 ng cFXIII/mg protein was measured. The synthesis of cFXIII by keratocytes was confirmed by RT-qPCR. Isopeptide cross-links were detected above, but not within the corneal stroma. Slight abnormality of the cornea was detected in six out of nine FXIII-A-deficient patients. The presence of cFXIII in human keratocytes was established for the first time. cFXIII might be involved in maintaining the stability of the cornea and in the corneal wound healing process.

## 1. Introduction

The transglutaminase (TG) family consists of nine members, eight of which blood coagulation factor XIII (FXIII) and TGs 1-7 are enzymatically active, while the enzymatically non-active erythrocyte band 4.2 protein also belongs to this group on the basis of structural similarity. TGs in their enzymatically active form (protein-glutamine:amine γ-glutamyltransferase; EC2.3.2.13) catalyze an acyl transfer reaction in which the carboxamide group of a peptide bound glutamine residue is the acyl donor and a primary amine is the acyl acceptor. If the primary amine happens to be the ε-amino group of a peptide bound lysine residue, the end result is the crosslinking of peptide chains by ε(γ-glutamyl)lysyl isopeptide bond. Although the sequential similarity among TGs is modest, their secondary structure is strikingly similar. All members of the TG family, including erythrocyte band 4.2 protein, contain an N-terminal β-sandwich domain, a central core domain with the active site, and two C-terminal β-barrel domains (for further details see references [1,2,3,4,5].)

FXIII is a unique TG, it is a coagulation factor present in the plasma playing an essential role in maintaining hemostasis by cross-linking and thereby stabilizing the fibrin clot. As opposed to all other TGs, plasma FXIII (pFXIII) is of tetrameric structure; it consists of two potentially active protransglutaminase A subunits (FXIII-A_2_) and two inhibitory/protective non-enzymatic B subunits (FXIII-B_2_). pFXIII becomes transformed into an active TG in the terminal phase of the coagulation cascade. Thrombin cleaves off a 36-mer peptide from the N-terminus of FXIII-A, then in the presence of Ca^2+^, the dissociation of FXIII-B relieves the inhibitory effect. In addition to being, the potentially active component of pFXIII, FXIII-A_2_ is also a cellular enzyme (cFXIII) present in a number of cell types. cFXIII was first discovered in platelets [6,7]; it amounts to 3% of the total platelet proteins [8]. cFXIII in resting platelets is of cytoplasmic localization [9,10], however activation by strong stimuli, like thrombin+collagen, can translocate part of it to the platelet surface [11]. In subsequent studies cFXIII was also demonstrated in monocytes/macrophages [12,13,14], in cells of mineralized tissue, i.e., in osteoblasts, osteoclasts, osteocytes [15,16], and most recently, in preadipocytes [17].

The occurrence of TGs in the eye was investigated by Raghunath et al. who detected FXIII in the endothelium of arterioles and capillaries in the conjunctival stroma [18]. In this study, the presence of cFXIII in the cornea was not explored. The human cornea proteome has been analyzed, and 3250 Swiss-Prot annotated proteins were identified by LC-MS/MS technique, among them a small amount of FXIII-A [19]. However, neither its distribution in the cornea nor its (intra)cellular localization has been explored. In a previous study, we investigated FXIII subunits in human tears in relation to penetrating keratoplasty [20]. FXIII-A concentration normalized for protein concentration increased 17-fold 24 h after the surgery. This finding raised the possibility that the extra cFXIII in the tears, at least in part, derived from the injured cornea, and prompted us to perform a comprehensive analysis of corneal cells for the presence and distribution of FXIII-A.

## 2. Results

### 2.1. Detection of FXIII-A in the Corneal Tissue by Immunohistochemistry and Western Blotting

**Figure 1:**
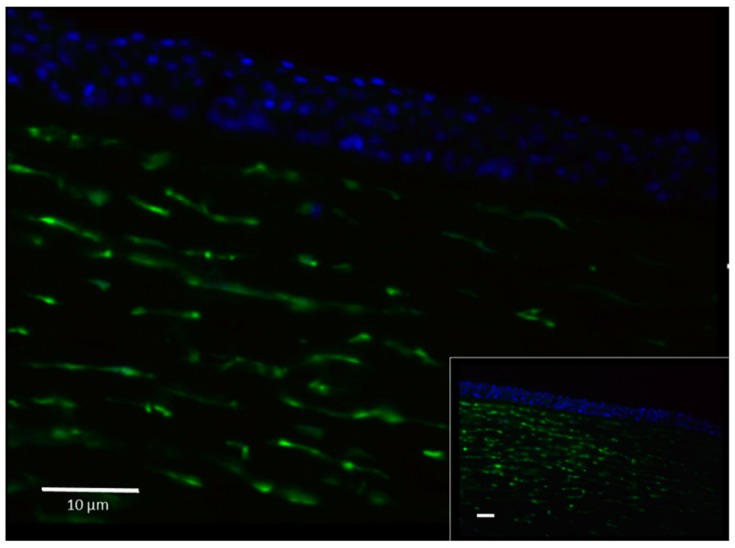
FXIII-A containing cells in the cross-section of the cornea at higher and lower (insert) magnification. FXIII-A-positive cells are shown in green. The section was counterstained by DAPI to demonstrate cell nuclei. Bars represent 10 μm.

**Figure 2:**
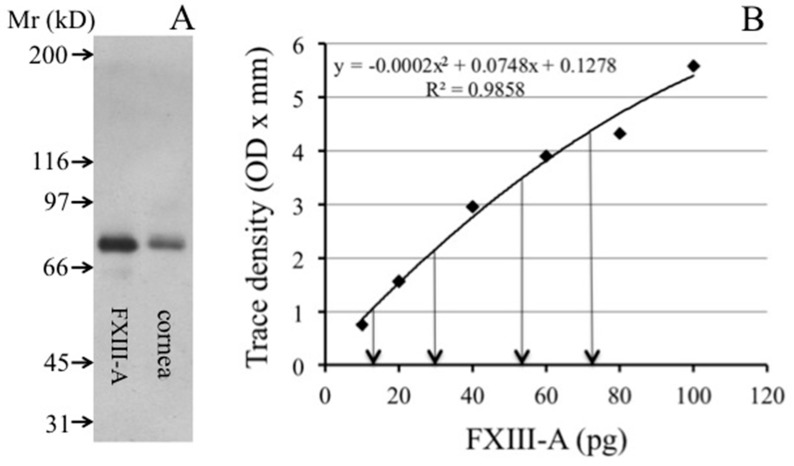
Detection and quantification of FXIII-A in the human cornea by Western blotting. (**A**) Demonstration of FXIII-A on the Western blot of cornea lysate. (**B**) Quantitative densitometric analysis of Western blots using FXIII-A standards and four different dilutions of a cornea lysate containing 3.5, 7, 14, and 28 μg corneal protein. The second-order polynomial equation that adequately describes the calibration curve is shown on the upper part of the figure.

The presence of FXIII-A in the cornea was first demonstrated by immunohistochemistry (Figure 1). Cells stained with an antibody specific for FXIII-A were abundant in the corneal stroma but absent in the epithelium (upper part of the section). In the cross-section of the cornea, FXIII-A-positive cells appear as elongated fusiform cells. No FXIII-B was detected in the cornea (not shown). The immunohistochemical finding was confirmed by Western blotting (Figure 2A). By quantitative densitometry, 3.64 (range: 2.69–4.47) ng FXIII-A/mg corneal protein was measured (Figure 2B).

### 2.2. The distribution of FXIII-A-Positive Keratocytes in the Corneal Stroma

**Figure 3:**
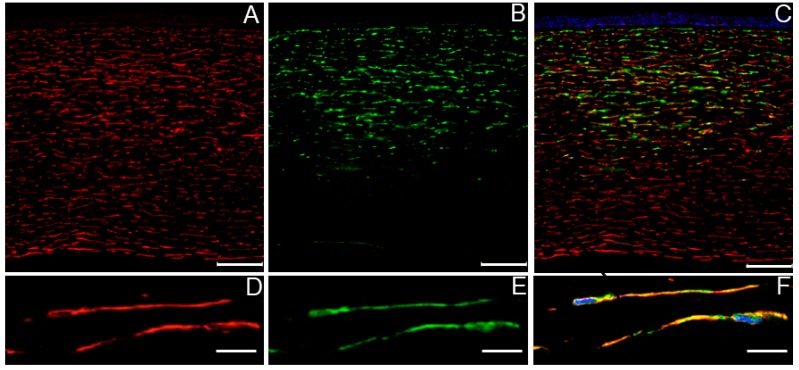
The distribution of CD34 positive keratocytes (**A**) and FXIII-A-positive cells (**B**) and their appearance in overlay picture (**C**) in a cornea section. In the lower part of the figure, two individual keratocytes were selected from the cross-section and shown at higher magnification. CD34 and FXIII-A positivity appear in red and green (**D**,**E**), respectively. The overlay picture (**F**) indicates their different distribution within the cell (DAPI labeled nuclei appear in blue). The scale bars represent 100 μm in A–C and 10 μm in D–F.

Figure 3A demonstrates that keratocytes labeled for CD34 were present throughout the corneal stroma. The mean cell count/visual field was 119 ± 5, 116 ± 6, and 115 ± 9 in the anterior, middle, and posterior tertile of the stroma, respectively. In contrast, FXIII-A-positive cells were more abundant in the anterior tertile of the stroma (Figure 3B). Their occurrence gradually decreased from the anterior to the posterior stroma, 120 ± 10, 72 ± 10, and 38 ± 6 cells/visual field were counted in the anterior, middle, and posterior tertile. Endothelial and epithelial cells lack FXIII-A. The overlay of the two staining patterns suggests that FXIII-A-positive cells are CD34 positive keratocytes (Figure 3C). This became even more evident when individual keratocytes enlarged from the cross-sectional picture were investigated for CD34 (Figure 3D) and FXIII-A (Figure 3E) positivity. The overlay picture of individual cells confirmed that the two antigens are in the same cells (Figure 3F), however, the separation of the green and red staining patterns suggest that CD34 and FXIII-A are located in different cell compartments.

### 2.3. FXIII-A Protein and FXIII-A mRNA in Isolated Keratocytes

**Figure 4:**
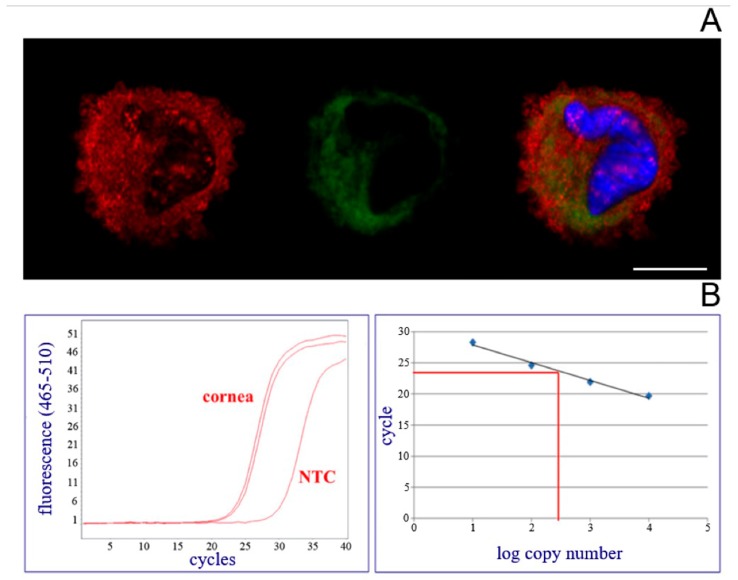
The presence of FXIII-A protein and mRNA in keratocytes isolated from human cornea. (**A**) In the upper part from left to right an isolated keratocyte stained for CD34 (red), FXIII-A (green), and the merged picture is shown (cell nucleus stained with DAPI appear in blue). CD34 appeared as a membrane protein, while FXIII-A is of cytoplasmic localization. Bar represents 10 μm. (**B**) Detection and quantitation of FXIII-A mRNA in keratocyte lysate by RT-qPCR. Left panel: Fluorescence acquired at each cycle is used for detection and quantification. NTC: No-template control; cornea: Technical replicate of one sample. Right panel: Standard curve on which quantification cycle was plotted against the log copy number of cDNA input.

Subsequently, individual keratocytes, isolated from the cornea lysate, were analyzed by immunostaining and flow cytometry. Their capacity for producing FXIII-A specific mRNA was also evaluated. In situ cornea keratocytes are large flat cells among collagen lamellae of the stroma and possess multiple contacts with neighboring cells [21,22]. In the cross-section of the cornea, they appeared as thin elongated cells. Isolated cells, however, lose their contact during the isolation procedure and their cytospin preparation showed rounded shape (Figure 4). The different location of CD34 and FXIII-A becomes evident by double immunofluorescence investigation (Figure 4A). CD34 is a membrane protein, while cFXIII is of cytoplasmic localization. The demonstration of FXIII-A mRNA by RT-qPCR in keratocytes (Figure 4B) strongly suggests that these cells are capable of synthesizing their own FXIII-A proteins.

### 2.4. Characterization of Isolated Corneal Keratocytes by Flow Cytometry

**Figure 5:**
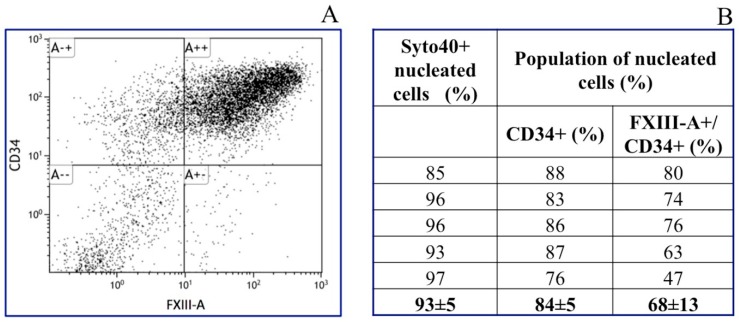
Flow cytometric analysis of isolated keratocytes. Nucleated particles were identified by Syto40 staining and they were characterized by staining for CD34 and FXIII-A. (**A**) The left panel shows a representative dot plot of CD34+ and FXIII+ cells prepared from a single cornea. (**B**) The right panel demonstrates the results obtained for cells from five individual cornea preparations. The contribution of CD34+ keratocytes to the total corneal cells and the frequency of FXIII-A+ cells in the keratocyte population were calculated.

Quantitative determination of FXIII-A containing cells among isolated keratocytes was carried out by flow cytometry. As shown in Figure 5, the great majority of nucleated corneal cells was positive for CD34, i.e., they represented keratocytes [23]. Approximately two-thirds of keratocytes were also stained for FXIII-A. This result is in accordance with the immunohistochemical finding and reveals that two populations of keratocytes (FXIII-A+ and FXIII-A-) exist in the human cornea.

### 2.5. Immunohistochemical Detection and Localization of Isopeptide Protein Cross-Links in the Cornea

**Figure 6:**
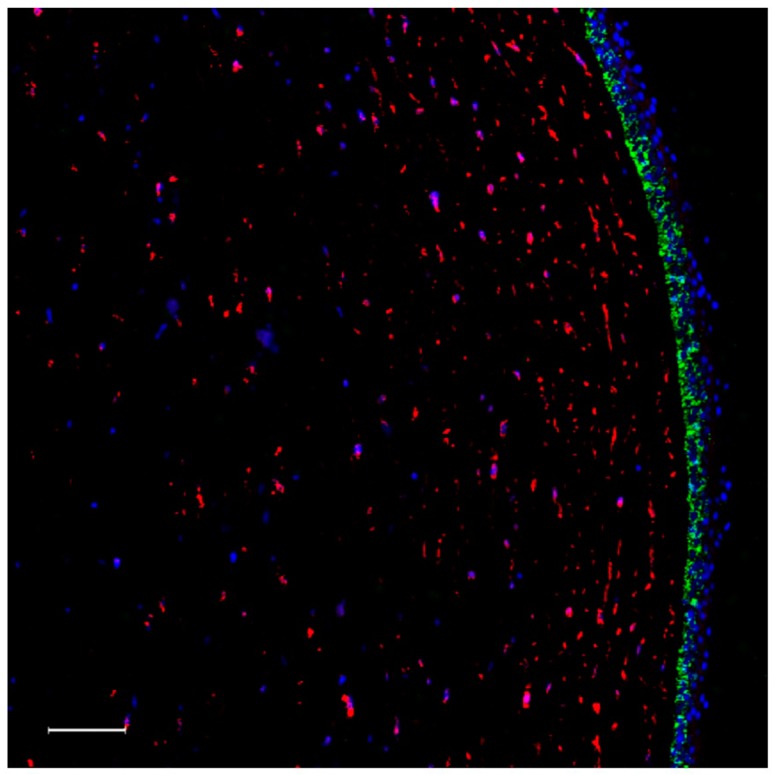
Immunohistochemical detection of ε(γ-glutamyl)lysyl isopeptide bonds in the human cornea. As opposed to the former sections, here the cross-section of the cornea is stained for FXIII-A in red. Isopeptide bonds are shown in green. Bar represents 100 μm.

The most general product of activated FXIII, just like that of most other TGs, is the cross-linking of proteins through ε(γ-glutamyl)lysyl isopeptide bonds. A specific antibody can reveal the localization of such bonds in tissue sections. In the cornea, this antibody did not stain the corneal stroma, but isopeptide bonds were revealed above the anterior stroma, very likely in part of the Bowman layer and the lower part of the epithelium (Figure 6).

**Figure 7:**
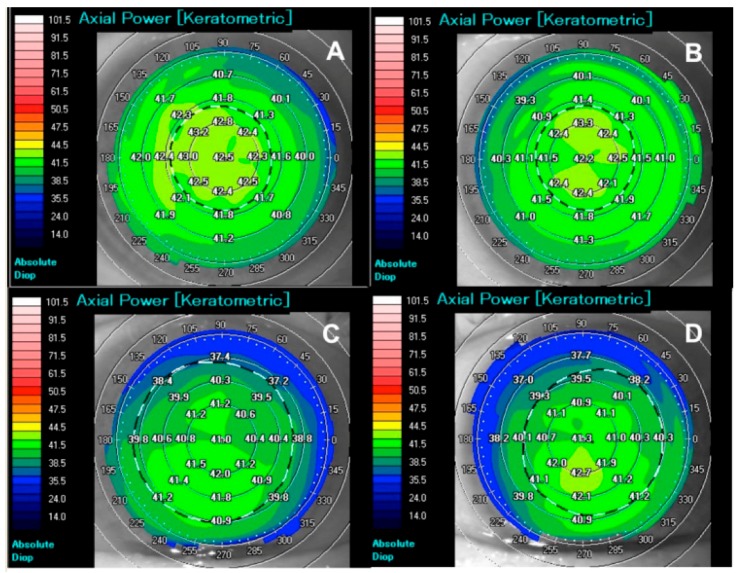
Representative corneal topographic pictures of two FXIII-A-deficient patients. (**A**) Normal spherical shape (right eye of patient 1 in Table 1), (**B**) symmetrical bow-tie shape (left eye of patient 1), (**C**,**D**) asymmetric bow-tie shape (right and left eye of patient 4). The numbers on the central part of the pictures show the keratometric values, the circles in white and black represent the size of the pupils. The pictures were taken by Tomey Casia anterior segment optical coherence tomography.

Comprehensive ophthalmological investigations were carried out to explore the presence of corneal abnormality in FXIII-A-deficient patients. One patient had cataracts on one eye, which caused slightly blurred vision (corrected distance visual acuity = 0.8), all other eyes had a visual acuity of at least 1.0. Slit lamp biomicroscopy showed no overt sign of keratoconus. Ultrasound and optical coherence tomography examinations excluded any rough ophthalmic diseases. Six out of nine patients had inferior steepening and asymmetric bow-tie pattern on the topographic picture on both eyes and 7 out of 18 eyes had abnormal inferior-superior diopter asymmetry (Table 1, Figure 7). One out of 18 eyes had astigmia higher than 1.5 D (patient 8). All other investigated parameters (central corneal power, skew of radial axis, corneal thickness at the thinnest point of the cornea, whether the thinnest point of cornea is outside of the central 2 mm, pachymetric difference between in the thinnest point and central average, back elevation) were in the range that would not raise suspicion for keratoconus.

## 3. Discussion

cFXIII is expressed in a few cell types, however its functions have been only partially explored. The potentially active subunit of pFXIII very likely derives from intracellular sources. Platelet FXIII could contribute to pFXIII [24], but most recently, resident macrophages have been identified as the major source of circulating FXIII-A [25]. In addition to being a source of pFXIII, cFXIII may exert its effect both in the intra- and extracellular compartment. For the latter, it needs to be released from the cells or to be translocated to the outer surface of the membrane. FXIII-A lacks the signal sequence and asparagine-linked glycosylation; consequently, it cannot be released through the classical secretory pathway. Although there is evidence that it can enter the alternative secretory pathway [26], as yet no direct proof has been provided on its release from the intracellular compartment by such a mechanism. Another way for the extracellular appearance of cFXIII is the translocation to the outer membrane surface of activated cells or the formation of cFXIII containing microparticles leaving the cells. The surface exposure of FXIII-A on cultured cells [16,17,27,28,29] and on activated platelets [11] has been demonstrated and microparticles released by activated platelets also contain cFXIII [30].

Obviously, cFXIII might also have functions in the intracellular compartment, although some of these functions are hard to separate from those exerted by the surface exposed molecule. The involvement of cFXIII in platelet spreading on fibrinogen as well as on collagen I-coated surfaces has been clearly demonstrated [31,32]. Although during platelet activation cFXIII becomes an active transglutaminase and cross-links contractile proteins [33,34,35,36], reports on its involvement in clot retraction are controversial and normal retraction could be elicited by FXIII-A-deficient platelets [37]. The impaired receptor mediated and non-receptor mediated phagocytosis by FXIII-A-deficient monocytes indicate that FXIII-A is involved in the mechanism of this monocyte function [38]. cFXIII is involved in the hypertrophic differentiation of chondrocytes [39] and contributes to the formation of calcium precipitates in the matrix of cultured cells [40]. In cooperation with other TGs, it contributes to the regulation of osteoclast differentiation [41]. cFXIII is also present in osteoblasts and osteocytes [42,43]. It was suggested that together with TG1 and TG2, cFXIII is a novel regulator of bone mass [44]. However, normal bone deposition was demonstrated in mice deficient both in FXIII-A and TG2 [45]. A more recent intriguing finding on the role of cFXIII concerns its presence in mouse preadipocytes and the demonstration that it acts as a negative regulator of adipogenesis as well as a promoter of preadipocyte proliferation [17]. More detailed accounts on the multiple roles of cFXIII and other TGs are provided in reviews [15,46,47,48,49].

In this study, the presence of FXIII-A in the cornea was confirmed both by immunohistochemistry and Western blotting. Neither the epithelium nor the endothelium showed FXIII-A positivity, while in the stroma, FXIII-A expressing cells were abundant. The contribution of FXIII-A to the total stromal protein is low, in the ng FXIII-A/mg corneal protein range. However, considering the overwhelming presence of collagen in the corneal stroma, this is not surprising. The detection of FXIII-A mRNA in the corneal cells clearly showed that FXIII-A is not coming from external sources; it is synthesized in situ. As demonstrated both by immunohistochemistry on corneal sections and by flow cytometry, FXIII-A+ cells also stained for CD34, a keratocyte marker [23]. Keratocytes represent the majority of stromal cells and by producing collagen and glycosaminoglycans they maintain the integrity of this layer [50]. Two-thirds of CD34+ cells were also positive for FXIII-A. Although the distribution of CD34+ cells was continuous in the corneal stroma, FXIII-A+ keratocytes tended to occur more frequently in the anterior part. For the time being, it is not clear if the two phenotypes of keratocytes (CD34+/FXIII-A- and CD34+/FXIII-A+) represent cell populations with functional differences.

Concerning the function of cFXIII and cFXIII+ keratocytes in the cornea only suggestions can be offered. cFXIII might exert its effect both in the intracellular and extracellular compartments. In isolated keratocytes, cFXIII was of cytoplasmic localization and, for the time being, it is not clear if it can be secreted or exposed to the surface. The main function of active pFXIII is to cross-link proteins. To explore such function in the cornea immunofluorescent staining of cornea sections with an antibody that specifically reacts with isopeptide bonds were performed. No protein cross-linking was detected in the stroma, which is not surprising because cross-linking of stromal proteins might impair corneal transparency. A layer of intensive staining for isopeptide bonds was detected in the lower part of or underneath the epithelium; the latter might include the Bowman layer. Such a layer of cross-linked proteins might be associated with the high mechanical stiffness of the Bowman layer and important for conferring structural stability to the cornea [51]. Such cross-linked substrate could also provide the niche for the transformation of limbal epithelial stem cells into differentiated epithelial cells [51]. It is interesting that the gradient of stiffness from the anterior (stiffer) to the posterior stroma (softer) [51] is similar to the differential distribution of cFXIII-positive keratocytes, however for the time being, no causal explanation can be offered. It is also an open question whether cFXIII present in stromal keratocytes could be released and by diffusing to the epithelial border could contribute to the cross-linked protein layer. Our first attempt to explore this possibility was to investigate the corneal topography of FXIII-A-deficient patients. No gross abnormality of the cornea was found in either of the patients. Slight irregularity, suggesting weakening of the cornea structure, was detected in six out of nine patients. The significance of this finding is unclear. However, it reminds us of the situation of impaired wound healing of FXIII-A-deficient patients. Only a fraction of these patients presented impaired wound healing [52,53], while in FXIII-A knock out mice, delayed and irregular healing of excisional wound was clearly manifested [54]. Evidently, keeping the structural integrity of the cornea, just like wound healing, is a complex process with multiple players, one of which could be cFXIII. Perhaps the investigations should be extended to other TGs. TGs, like TG1, TG2, and TG3, might also play a role in the formation of cross-linked corneal structures and in the structural stabilization of the cornea.

In this study, only quiescent keratocytes were investigated. We believe that our results open up a new area of investigation on the involvement of cFXIII in corneal pathology. In pathological conditions, like injury or infection, transdifferentiated keratocyte showed drastic changes: They became capable of proliferation and migration, they produced adhesive proteins, and expressed contractile myofibroblast character [22]. cFXIII might be involved in these changes, and it would also be interesting to explore if FXIII+ and FXIII- keratocytes behave differently. The role of cFXIII in corneal wound healing seems to be a particularly promising topic. Cultured corneal epithelial stem cells proliferated and formed colonies in fibrin gel cross-linked by plasma FXIII present in cryoprecipitate [55]. Dardik et al. demonstrated that a considerable amount of FXIIIa injected into the cornea exhibited a proangiogenic activity and induced vascularization [56]. It is well possible that in the case of corneal injury, involving the stroma, keratocyte cFXIII becomes externalized and depending on its concentration, improves the healing process or, at a higher concentration, is involved in the process of undesired vascularization. In our former study, it was shown that after penetrating keratoplasty, patients who developed neovascularization of donor cornea had considerably higher pre- and post-surgery FXIII levels in tears than patients with non-neovascularized cornea [20].

The results presented above clearly demonstrate cFXIII in part of CD34+ corneal keratocytes. These cells are capable of synthesizing cFXIII and they are concentrated in the anterior corneal stroma. They might be involved in the formation of an isopeptide-rich layer below and in the lower part of epithelium and also could contribute to the structural stability of the cornea. The results open up new directions in the cornea research, part of which we have started pursuing. The presence and cellular localization of other TGs and their relationship to the isopeptide-rich protein layer is also of considerable interest. Extension of the studies to the limbal region could also provide important pieces of information. Experiments with isolated keratocytes could shed lite on intracellular trafficking, mechanism of extracellular release of cFXIII, etc. Understanding the mechanism of corneal wound healing could also profit from studying the involvement of cFXIII containing keratocytes in this process.

## 4. Materials and Method

### 4.1. Cornea Samples

Sixteen corneas from nine donors (age 43–85 years, mean: 62.7) were used for the investigations. Two cornea samples were obtained from eyes enucleated due to retrobulbar melanoma. The corneas were removed immediately after enucleation. Other cornea samples were obtained from cadavers. For the inclusion of the latter cornea samples in the study, the same criteria were used as for corneas removed for corneal transplantation. The cornea samples were removed within 12 h of biological death. The cause of death was neoplasm without cerebral metastasis in five cases and ischemic heart disease in two cases. None of them suffered any kind of eye disease, HIV, hepatitis B and C, or bacterial infection. A history of former eye surgery was also an exclusion criterion. Ethical permission for the study of cornea samples was obtained from the Regional Ethics Committee of the Medical Faculty, University of Debrecen, Hungary, and approved by the Office of the Chief Medical Officer for Hungary, Budapest.

### 4.2. FXIII Deficient Patients

Two Hungarian, five Finnish, and two Swiss FXIII-A-deficient patients (4 females, 5 males, age 18–63) were recruited for non-invasive ophthalmological examinations (visual acuity, slit lamp examination, corneal topography, ultrasound, and optical coherence tomography). The investigations were approved by the respective national ethics committees, by the Office of the Chief Medical Officer for Hungary, by the Research Ethics Committee of the Faculty of *Medicine*, University of Helsinki, Finland and by the Kantonale Ethikkommission, Bern, Switzerland. FXIII-A-deficient patients who underwent noninvasive ophthalmological investigations gave informed consent. The diagnosis of severe FXIII-A deficiency was established by measurement of FXIII activity in patients’ plasma and confirmed by identifying the mutations in the *F13A1* gene. All patients had causative mutations, in homozygous or in double heterozygous form (see details in Table 1). At the time of investigation, FXIII-A-deficient patients were on prophylaxis using plasma-derived FXIII concentrate (Fibrogammin P/Cluvot, CLS Behring, Marburg, Germany) for the Finnish and the Swiss patients or recombinant FXIII-A_2_ (Novothirteen, Novo Nordisk A/S, Bagsvaerd, Denmark) for the Hungarian patients.

### 4.3. Immunohistochemistry

Cornea samples for immunohistochemistry were embedded in Shandon Cryomatrix freezing medium (Thermo Scientific, Waltham, MA, USA) and stored at −80 °C. Frozen sections (7 µm) were fixed with acetone and incubated with normal human serum diluted 6-fold in PBS to prevent non-specific IgG binding. Then, sections were incubated with one of the following antibodies: Monoclonal mouse antibody against FXIII-A produced in our laboratory [8], polyclonal rabbit anti-FXIII-A (Dade Behring, Marburg, Germany), or polyclonal rabbit anti-FXIII-B (Sigma, St. Louis, MO, USA). Immune-labeling was visualized by either FITC-labeled anti-mouse or FITC-labeled anti-rabbit antibodies produced in goat (Vector Labs, Burlingame, CA, USA). For double labeling immunoreactions, slides were first incubated with rabbit anti-human FXIII-A antibody, then with FITC conjugated goat anti-rabbit IgG. CD34 antigen was detected by incubation with monoclonal anti-human CD34 antibody (Abcam, Cambridge, UK). The reaction was visualized by biotinylated horse anti-mouse IgG (Vector Labs) followed by Texas Red-labeled streptavidin (Vector Labs). For the labeling of isopeptide bonds, IgM type mouse monoclonal antibody from Covalab (Villeurbanne, France) was used and visualized by Alexa Fluor 488 conjugated goat anti-mouse IgM antibody (Abcam, Cambridge, UK). In this case, counterstaining for FXIII-A was carried out with Texas Red conjugated goat anti-rabbit antibody (Vector Labs, Burlingame, CA, USA). Vectashield mounting medium with DAPI (Vector Labs) was used for mounting slides, to counterstain nuclei and avoid bleaching fluorescence. All reactions were carried out at room temperature; phosphate buffered saline was used for the dilution of antibodies and in washing steps. For negative controls, the primary antibodies were replaced by the respective non-immune control sera.

Slides were investigated with an Axioplan fluorescence microscope (Carl Zeiss Obekochen, Germany) equipped with selective filters and connected to CCD IMAC camera (Sony, Tokyo, Japan) plus ISIS fluorescent imaging system (Metasystems, Altlussheim, Germany). Representative images were acquired by confocal laser scanning microscope (LSM 700, Zeiss Oberkochen, Germany) equipped with Plan-Apochromat 63x/1.40 oil objective and solid-state lasers. Separation of the fluorescence signals was performed by selective laser excitation (405 nm, 488 nm, 555 nm laser lines) coupled to efficient splitting of the emission using variable secondary dichroic beam-splitter.

### 4.4. Western Blotting

Epithelium and endothelium were removed by blunt knife; the remaining stroma was cut into small pieces and transferred into SDS PAGE sample buffer containing 8 M urea. The samples were homogenized by sonication, then denatured in boiling water for 5 min, and finally subjected to continuous shaking for 2 days. After centrifugation, the total protein concentration of the supernatant was measured by BCA protein assay kit (Pierce, Rockford, IL, USA). After reduction by 5% 2-mercaptoethanol, the denatured proteins were separated by SDS PAGE (7.5% gel) and electro-transferred to PVDF membrane. FXIII-A was detected by affinity purified sheep anti-FXIII-A antibody (Affinity Biologicals, Ancaster, Canada) followed by biotinylated anti-sheep IgG and avidin-biotinylated peroxidase complex (components of Vectastain ABC kit; Vector Labs). The immunoreaction was visualized by enhanced chemiluminescence detection (ECL Plus+, Amersham, Little Chalfont, UK) according to the manufacturer’s instructions. Biotinylated SDS PAGE standard (Bio-Rad, Hercules, CA, USA) was used for the estimation of molecular mass. The amount of FXIII-A in the lysate of corneal stroma was determined by quantitative densitometric analysis of the Western blot. A standard curve was created using different concentrations of highly purified FXIII-A_2_ [57]. In the case of each cornea sample, determinations were carried out in quadruplicate using 4 different dilutions. The differences between individual results were less than 6%. The mean and the range of the results obtained from 3 cornea samples were calculated.

### 4.5. Isolation of Keratocytes from the Cornea and Their Immunofluorescent Analysis

Five de-epithelized and de-endothelized corneas were used for keratocyte preparation. Each cornea was inserted into 500 μL Hank’s Balanced Salt Solution 1x (Gibco, Carlsbad, CA, USA) containing 2 mg Collagenase I (Gibco) and incubated for 4 h at 37 °C under constant agitation. Cytospin preparations of the cells were used for double immunofluorescent analysis. After fixation with 20% acetone, staining for FXIII-A and CD 34 was carried out as described for tissue sections.

### 4.6. Detection and Quantification of FXIII-A mRNA in Keratocytes

Total RNA was prepared from keratocytes isolated from four corneas using High Pure RNA Tissue Kit (Roche, Mannheim, Germany). Reverse transcription was performed on 50 ng of the isolated total RNA in 20 µL by Transcriptor First Strand cDNA Synthesis Kit (Roche) according to the manufacturer’s instructions. For the detection and analytical evaluation of FXIII-A mRNA by real-time RT-qPCR, specific PCR amplicon of the gene was used as standard. A 116 base-pair fragment was amplified using 5′-TCACGAGCGTTCACCTGTTC-3′ forward primer and 5′-CTGCACATAGAAAGACTGCCC-3′ reverse primer with 60 °C annealing temperature. Specific PCR products were separated by 1% agarose gel electrophoresis, excised, and purified (High Pure PCR Cleanup Micro Kit; Roche). The concentration of the purified nucleic acid was calculated by measuring the absorbance at 260 nm and converted into copies per microliter [58]. Tenfold serial dilutions of the quantified FXIII-A amplicons (range: 10–10^5^ copies/μL) were used as external standards for the FXIII-A real-time PCR reaction. The calibration curve used for FXIII-A amplicon number calculation was created by plotting the quantification cycle (Cq) corresponding to each standard versus the value of the respective log number of FXIII-A amplicon concentration. PCR efficiency was determined using four serial 2-fold dilution points; Cq(s) were plotted versus the logarithm of dilution [59]. Efficiency for used primer pair was 112%. SPUD assay demonstrated absence of inhibition in each qPCR reaction [60].

### 4.7. Flow Cytometric Analysis of Isolated Keratocytes

Cells from the lysed cornea were pelleted by centrifugation and resuspended in phosphate buffered saline (PBS). Keratocytes suspensions were counted in Bürker chamber and also analyzed on Sysmex XN-350 L hematology analyzer (Sysmex Europe GmbH, Germany). Cell counts of 10^6^–10^7^ could repeatedly be harvested by the previously described isolation procedure. In a typical experimental setting, we stained 10^5^ cells for flow cytometric analysis.

Staining was carried out in the dark at room temperature. First, cells were incubated with antiCD34-PerCPCy5.5 antibody (Becton Dickinson Pharmingen, San Jose, CA, USA) for 20 min. After this surface staining, cells were immediately fixed by IntraStain A solution (Dako, Glostrup, Denmark) for 15 min. Subsequently, cells were washed once in PBS and resuspended in IntraStain B solution and simultaneously stained by FITC conjugated anti FXIII-A antibody or with FITC-conjugated non-immune mouse IgG for 20 min. Upon completion, cells were washed once in PBS and stored in 1% paraformaldehyde. Immediately before flow cytometric analysis, cell nuclei were stained by Syto 40 (Invitrogen ^TM^, Thermo Fischer Scientific, Rockford, IL, USA).

### 4.8. Non-Invasive Ophthalmological Examinations

Each FXIII patient underwent comprehensive ophthalmologic examination, including a review of their medical history, uncorrected and corrected distance visual acuity measured by decimal notation, manifest refraction, slit lamp biomicroscopy, fundus examination with a +90D non-contact lens, ultrasound, and corneal topography. Tomographic parameters such as parameters from the front and back cornea were measured in 4 cases by Pentacam (OCULUS Gmbh, Wetzlar, Germany), and in 5 cases by Tomey Casia 2 (TOMEY Corp, Nagoya, Japan). Parameters retained for the analysis were keratometry readings; topographic astigmatism and asphericity for the anterior and posterior corneal surfaces; pachymetry; topometric indices; and data from corneal thickness spatial profiles. Corneal thickness is defined as the thinnest point in the corneal thickness map. Belin–Ambrosio-enhanced ectasia display was also used in the case of Pentacam investigations. For elevation data measurement, the best-fit sphere served as a reference body using the float option; the diameter of the reference surface was 8 mm. The following abnormal parameters were considered as suspicious for keratoconus: Central corneal power > 47.2 D, inferior–superior diopter asymmetry >1.2, simulated keratometry astigmia > 1.5 D, skew of radial axis >21°, difference between the thinnest and median corneal thickness > 63 μm, corneal thickness on the thinnest point of the cornea < 492 μm, and thinnest point of cornea is out of the central 2 mm area [61,62].

## Figures and Tables

**Table 1:** Cornea parameters of FXIII-A-deficient patients.

Pts	Age	Gender	Mutations	Inferior-Superior Diopter Asymmetry		Inferior Steepening, Asymmetric Bow-Tie Pattern by Topography	
				OD	OS	OD	OS
**1**	**60**	**F**	IVS5(-1) G>A	0.0	0.4	−	−
2	62	M	IVS5(-1) G>A	0.4	0.8	−	−
3	20	M	p.R662Y	0.4	0.2	+	+
4	55	F	p.R662S	**1.5**	**2.6**	+	+
5	57	F	*	**1.8**	0.2	**−**	**−**
6	36	F	p.M159R	0.7	1.0	**+**	**+**
7	60	M	p.M159R p.R661X	**1.9**	**1.4**	**+**	**+**
8	22	M	p.R382S p.Q400X	**1.4**	**1.3**	**+**	**+**
9	18	M	IVS5 (-1) G>A	0.5	0.6	**+**	**+**

Measurements and observations on the right (OD) and left (OS) eye are shown separately. Pts: Patients, F: Female, M: Male. −, + without and with cornea irregularity, respectively. * Patient 5 is the sister of patient 4, genetic investigation only identified the lacking domain. Bold setting indicates data that could raise the suspicion for keratoconus.

## Abbreviations

Cq	quantification cycle
FXIII	blood coagulation factor XIII
FXIII-A	FXIII A subunit
FXIII-B	FXIII B subunit
cFXIII	cellular FXIII
pFXIII	plasma FXIII
PBS	phosphate buffered saline
TG	transglutaminase
